# Brain Structural and Functional Neuroimaging Features are Associated With Improved Auditory Hallucinations in Patients With Schizophrenia After Real-Time fMRI Neurofeedback

**DOI:** 10.1155/da/2848929

**Published:** 2025-04-08

**Authors:** Jiahe Zhang, Emma Tusuzian, Francesca Morfini, Clemens C. C. Bauer, Lena Stone, Angelina Awad, Ann K. Shinn, Margaret A. Niznikiewicz, Susan Whitfield-Gabrieli

**Affiliations:** ^1^Department of Psychology, Northeastern University, Boston 02115, Massachusetts, USA; ^2^Department of Psychiatry, Massachusetts General Hospital, Boston 02114, Massachusetts, USA; ^3^Department of Psychiatry, Harvard Medical School, Boston 02115, Massachusetts, USA; ^4^Department of Brain and Cognitive Sciences and McGovern Institute for Brain Research, Massachusetts Institute of Technology, Cambridge 02139, Massachusetts, USA; ^5^Psychotic Disorders Division, McLean Hospital, Belmont 02478, Massachusetts, USA; ^6^Department of Psychiatry, Boston VA Healthcare System, Boston 02130, Massachusetts, USA; ^7^Department of Psychiatry, Boston VA Research Institute, Boston 02111, Massachusetts, USA

## Abstract

Auditory hallucinations (AHs) are debilitating and often treatment-resistant symptoms of schizophrenia (SZ). Real-time functional magnetic resonance imaging (fMRI) neurofeedback (NFB) is emerging as a flexible brain circuit-based tool for targeting AH via self-modulation of brain activity. A better understanding of what baseline characteristics predict NFB success will enhance its clinical utility. Previous work suggests that AH symptomology implicates measures across multiple modalities, including T1 structural MRI (sMRI), diffusion-weighted MRI (dMRI), and resting-state fMRI (rsfMRI). Specifically, AH severity and treatment response are associated with thinner superior temporal gyrus (STG), thinner dorsolateral prefrontal cortex (DLPFC), reduced white matter integrity in tracts connecting brain regions implicated in SZ symptomatology, increased within-default mode network (DMN) connectivity, and reduced DMN–DLPFC anticorrelation. In this study, we tested the individual and combined contributions of multimodal brain features for the prediction of AH change after NFB in adults (*N* = 25, 36.1 ± 10.0 years, 24% females) with SZ spectrum disorders (SZ or schizoaffective disorder) and frequent medication-resistant AH. Participants underwent a baseline MRI scan (including sMRI, dMRI, and rsfMRI) and were randomly assigned to receive NFB from their STG (*n* = 12, real condition) or NFB from their motor cortex (MC) (*n* = 13, sham condition). NFB success was operationalized as the improvement in AH severity after NFB. We found that higher baseline AH severity, greater STG thickness, decreased dorsal cingulum integrity, increased within-DMN resting-state functional connectivity, and increased DMN–DLPFC anticorrelation were each individually correlated with reduction in AH severity. However, in a combined regression model, DMN–DLPFC connectivity emerged as the only independent variable that explained the unique variance in AH change. These results suggest that a specific rsfMRI measure, namely DMN–DLPFC connectivity, may be a promising predictor of NFB success in reducing AH and support the precision medicine approach.

**Trial Registration:** ClinicalTrials.gov identifier: NCT03504579

## 1. Introduction

Auditory hallucinations (AHs) are among the most debilitating symptoms of schizophrenia (SZ), with about 75% of patients diagnosed with SZ suffering from AH [1]. AH are heterogeneous, encompassing single or multiple voices that can be familiar or unfamiliar, speaking sequentially or simultaneously, and giving commands, comments, insults, or encouragement [2]. AH is a persisting burden for many patients [3] and is often associated with reduced work attainment and worse prognosis [4]. However, AH is often difficult to treat, with about one-third of individuals with SZ experiencing pharmacology-resistant AH [5, 6]. Psychotherapy, such as cognitive behavioral therapy, is often paired with pharmacology to help cope with the emotional distress associated with AH [7]. However, it may not improve the severity [7] or frequency [6] of AH. Recent neuromodulatory methods such as transcranial magnetic stimulation or electroconvulsive therapy may not be well tolerated and may be limited in effectiveness [8, 9]. AH has been associated with low treatment efficacy, likely due to individual differences in treatment responses, so it is highly important to develop novel, noninvasive, and personalized treatments to reduce the severity and frequency of AH.

Increasingly, researchers are hypothesizing that AH arises from abnormalities in a network of brain regions. Patients may misattribute the source of personal inner thoughts to others, which has been explained by aberrant interactions between prefrontal and temporal regions [10, 11]. Models of AH often implicate the superior temporal gyrus (STG), an auditory processing area, as a central region associated with this symptom. For example, hyperactivation of the temporal cortex in regions such as the STG is associated with AH [12, 13]. Further, the default mode network (DMN), composed of midline hubs in the medial prefrontal cortex (MPFC) and posterior cingulate cortex (PCC), is implicated in altered self-referential processing and shows hyperactivation and hyperconnectivity in SZ [14, 15]. It has been theorized that hyperconnectivity between the auditory cortex and the DMN underlies AH [16, 17].

Real-time functional magnetic resonance imaging (fMRI) neurofeedback (NFB) has emerged as a novel brain circuit-based intervention that allows self-modulation of brain activity while receiving feedback on that region's activity [18, 19], with applications in conditions including chronic pain [20], depression [21], and Parkinson's disease [22]. Specifically for SZ, some studies have used STG-directed real-time fMRI NFB to modulate the activity of AH-linked brain regions, which resulted in associated AH change [23, 24]. More recently, our group combined fMRI NFB with a specific strategy, mindfulness meditation, to help patients with SZ and pharmacology-resistant AH redirect attention away from auditory signals and reduce STG activity [25].

Given the heterogeneity in SZ symptomology and the high cost of fMRI, as well as in line with the precision medicine approach in psychiatry [26], it is crucial to know which patients may most benefit from NFB intervention. This can be achieved by identifying predictors of NFB success. We can examine initial symptom severity as well as capitalize on baseline neuroimaging characteristics such as cortical thickness from T1 structural MRI (sMRI), fractional anisotropy (FA) of white matter from diffusion-weighted MRI (dMRI), and functional connectivity from resting-state fMRI (rsfMRI). Thinning in key areas in processing and comprehending auditory input, such as the primary auditory cortices in the STG (see review in [27]), is associated with AH symptom severity [28–30] and predicts antipsychotic treatment response [31]. Further, the dorsolateral prefrontal cortex (DLPFC), a region associated with cognitive skills such as attention, working memory, and processing speed, shows thinning in patients with SZ [27, 32] that is associated with cognitive impairment [33] and treatment resistance [34].

Extensive AH research using dMRI has revealed reduced structural integrity in white matter tracts connecting regions of the DMN, STG, and DLPFC, such as the arcuate fasciculus (AF; [35–38]), superior longitudinal fasciculus (SLF; [39–41]), the genu and body of the corpus callosum (CC; [35, 40, 41]), and the cingulum [35, 36, 41, 42] in patients with SZ. However, both positive [38, 39, 43, 44] and negative [35, 36, 42, 45] correlations have been reported between FA and AH severity. Importantly, several studies showed that lower baseline integrity of the SLF, CC, and cingulum is associated with better response to antipsychotic treatment [46–48].

Resting-state functional connectivity within the DMN is aberrant in individuals with SZ [15] and is thought to contribute to the misattribution of internal thoughts to an external source in those with AH [49, 50]. DMN–DLPFC anticorrelation is also reduced in SZ [15, 51] and is linked to cognitive impairment [52]. Previous studies have identified within-DMN connectivity and DMN–central executive network (CEN, of which DLPFC is a canonical node) connectivity as predictors of treatment response in SZ [53–55], suggesting they could predict NFB success as well.

In this study, we leveraged multimodal brain features to investigate potential associations with reductions in AH. Twenty-five participants with SZ or schizoaffective disorder and medication-resistant AH underwent an fMRI NFB session in which they were instructed to either upregulate brain activity while listening to their own voice or downregulate brain activity while ignoring a stranger's voice. During NFB, participants randomized to the real condition (*n* = 12) were given visual feedback of their STG activation, and participants randomized to the sham condition (*n* = 13) were given visual feedback of their motor cortex (MC) activation. Other analyses based on this research project have reported reduced STG activation while listening to sentences recorded in the participant's own voice [56], increased DMN activation during self-referential processing [57], and reduced resting-state DMN and STG functional connectivity [58]. NFB success was defined by improvement on an AH scale (psychotic symptom rating scales; PSYRATS). We hypothesized that larger reductions in AH (i.e., symptom improvement) would be associated with brain functional and structural metrics that are associated with greater symptom severity and/or preserved cognitive abilities. Specifically, we hypothesized that (1) reduced STG thickness, (2) reduced DLPFC thickness, (3) reduced integrity in white matter tracts connecting regions involved in AH (AF, SLF, CC, and cingulum), (4) increased within-DMN connectivity, and (5) increased DMN–DLPFC anticorrelation would be associated with more symptom improvement.

## 2. Methods

### 2.1. Participants

Forty-one patients were enrolled in this study. Eligibility criteria included to (i) be diagnosed with SZ or schizoaffective disorder (DSM-IV) established by a clinician using the Structured Clinical Interview for DSM-5 (SCID); (ii) be experiencing moderate or severe medication-resistant AVH at least three times a week in the past month (positive and negative syndrome scale item #3 ≥ 4); (iii) be 18–55 years old; (iv) be on stable atypical neuroleptics medications dosage; (v) be right-handed (Edinburgh Handedness Inventory ≥ 60); (vi) be English native speakers; (vii) have estimated IQ ≥ 80 (WASI); (viii) have normal or corrected-to-normal vision and normal hearing; and (ix) be able to undergo MRI scanning. Participants were excluded if they (i) had a history of neurological or traumatic head injury in the past 6 months; (ii) had alcohol use disorder or substance use disorder in the past month (as per DSM-5), or used alcohol in the previous 24 hours; (iii) were pregnant.

All participants gave written consent obtained in accordance with the guidelines by Institutional Review Boards of Harvard Medical School, Boston VA Healthcare System, Massachusetts Institute of Technology (MIT), and Northeastern University (NEU; IRB#18-0324). Six participants were deemed ineligible due to exclusion criteria after enrollment. Five participants withdrew during the course of the study. Four participants did not have complete data as they were lost to contact during the course of the study. This resulted in a final sample of 25 participants (36.1 ± 10.0 years; 24–54 years; 24% females) who were randomly assigned to receive either real NFB (*n* = 12) or sham NFB (*n* = 13) (detailed demographic and clinical characteristics can be found in [57]. Two participants were lost to contact following the NFB session and, therefore, had missing post-NFB AHs scores.

### 2.2. MRI Acquisition

During the course of our study, we were required to change sites and scanners. Among the 25 participants, seven were scanned on a Siemens Trio scanner at MIT (32-channel), four were scanned on a Siemens Prisma scanner at MIT (32-channel), and 14 were scanned on a Siemens Prisma scanner at NEU (64-channel). On the Trio, anatomical data were acquired using a T1-weighted magnetization-prepared rapid acquisition gradient-echo (T1-MPRAGE) pulse sequence (1 mm isotropic, TR = 2530 ms, TE = 1.61 ms, FA = 7°, TI = 1200 ms) and functional data were acquired using a gradient-echo, echo-planar imaging (EPI) pulse sequence (3.5 mm isotropic, TR = 2000 ms, TE = 30 ms, FA = 90°). On the Prisma, anatomical data were acquired using a T1-MPRAGE (1 mm isotropic at MIT or 0.8 mm isotropic at NEU, TR = 2530 ms, TE = 1.7 ms, FA = 7°, TI = 1.4) and functional data were acquired using EPI (2 mm isotropic, TR = 1.2 s, TE = 30 ms, FA = 72°).

### 2.3. AH Assessment

Hallucinatory experience (AH score) was captured using the PSYRATS [59]. The PSYRATS includes 11 items measuring frequency, duration, controllability, loudness, location, disruption, distress (severity and intensity), negative content (amount and degree), and beliefs about the origin of voices on a scale of 0 (absent) to 4 (severe), where higher PSYRATS scores indicate more severe AH [60].

### 2.4. Session 1

#### 2.4.1. AH Assessment

Participants were administered the PSYRATS at the beginning of the session.

#### 2.4.2. STG Functional Localizer

Individual participant STGs were defined through a functional localizer task in which participants listened to previously recorded sentences. They were played a total of 80 unique sentences, with 40 sentences read in their own voice and 40 sentences read in a stranger's voice. The task involved three conditions: self-voice, other-voice, and rest. There were five sentences each in the self-voice and other-voice blocks, the former consisting of unique sentences spoken in the participant's voice and the latter including unique sentences spoken in a stranger's voice. During rest blocks, participants were presented with a crosshair on the screen. To sustain attention, for the last sentence of each self-voice and other-voice block, participants were instructed to answer a “yes” or “no” question regarding the content of the preceding sentence. At the end of each rest block, participants were instructed to answer a “yes” or “no” real-world question. Each run contained 12 pseudo-randomized blocks composed of eight self-voice, other-voice, and rest blocks across the two runs. Pseudo-randomization prevented rest blocks from directly following each other. A participant's individual target STG was created from the cluster containing the maximum intensity voxels in bilateral STGs (bilateral anterior and posterior STG regions of the Harvard-Oxford 25% probability atlas) after thresholding for the contrast Self-block >Other-block. The statistical threshold was varied in order to keep the functionally defined ROI a similar size (average = 190.1 voxels, SD = 35.94) across participants.

#### 2.4.3. DMN and MC Functional Localizer

We localized each participant's DMN and MC using rsfMRI following the procedure detailed in [61]. We used the signal from the MC in the sham condition since signal fluctuations in the MC are likely unrelated to hallucinations [11, 62]. First, the rsfMRI data were motion corrected, skullstripped, coregistered, smoothed, and bandpass filtered FSL 6.0 [63]. We then conducted an independent components analysis (ICA) using Melodic ICA version 3.14 [63] and extracted 30 spatiotemporal components. For each subject, we selected the component that most correlated with a standard DMN and motor networks [64] using FSL's “fslcc” tool and binarized the top-loading voxels (10%) to obtain the masks. A trained experimenter visually inspected the mask to ensure satisfactory coverage of the canonical DMN and MC.

### 2.5. Session 2

Session 2 occurred 10.0 ± 10.2 days after Session 1. In Session 2, participants completed: mindfulness training, pre-NFB rsfMRI, NFB, and post-NFB rsfMRI.

#### 2.5.1. Mindfulness Training

Participants were trained on a mindfulness technique called “mental noting,” a type of insight meditation practice [65] that emphasizes “concentration” and “observing sensory experience.” They learned to become aware of sensory experiences without engaging in their details by simply making a “note” of the most salient sensory modality (e.g., “hearing,” “seeing,” “feeling”) every second. Participants also learned to use an “anchor,” or a sensory experience that they could easily direct their attention towards (e.g., breathing) when they noted consecutive “hearing.” Participants practiced noting out loud to demonstrate their “noting” ability.

#### 2.5.2. NFB

**Figure 1:**
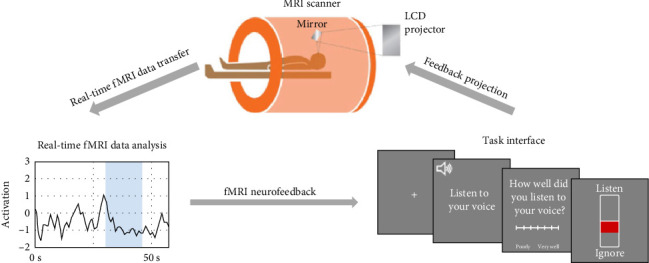
Mindfulness-based neurofeedback experiment setup. MRI scanner: The task display was projected and reflected in a mirror attached to the scanner head coil. Real-time fMRI data analysis: During the task, fMRI data were transferred and analyzed to determine the STG activation level when voice recordings were played. On neurofeedback runs, the activation level was displayed within the task interface. Task interface: Participants were shown a cue to either attend to their own voice or ignore a stranger's voice. Participants were then played the corresponding voice recordings. Participants were asked to provide a self-rating on their listening performance. On neurofeedback runs, activation level was displayed on a thermometer, indicating how well they attended to their voice or ignored a stranger's voice. fMRI, functional magnetic resonance imaging; MRI, magnetic resonance imaging; STG, superior temporal gyrus.

During the NFB task, participants were played a total of 80 sentences across six functional runs, with 40 recorded in their own voice and 40 recorded in a stranger's voice. Participants were given NFB during runs 2–5 but not in runs 1 and 6. Each run consisted of four randomized blocks, with two being “listen” and two being “ignore” blocks. Each block was composed of three sentences repeated twice and lasted 16 s. In the “listen” blocks, participants were told to attend to their own voices. In the “ignore” blocks, participants were instructed to move their attention away from the stranger's voice and all other scanner sounds by performing the mental noting taught during the mindfulness training immediately before the NFB task. A cue was presented at the beginning of each block to inform them of the block type. In the NFB runs, participants were shown an image of a thermometer after each block that indicated how well they attended to their voice or ignored all sounds based on their STG activation during each block type. The thermometer had a green or red bar, and its height on the thermometer was determined by the participant's STG activation level during the block. It was green if participants successfully upregulated their STG during the listening task and downregulated their STG during the ignore task. It was red if participants did not successfully regulate their STG activation in these desired directions. They were told the thermometer would display a green color if they successfully attended to their voice or ignored all sounds and a red color if they did not successfully attend to their voice or ignore all sounds. We provided real-time feedback of the participants' STG activation (Figure 1).

The displayed STG activity was the mean activity across all voxels within each participant's STG mask (as defined by 2.4.2.: *STG Functional Localizer*) estimated in real-time [66]. In each run of the NFB task, the first 30 s served as a baseline for an incremental general linear model (GLM) that incorporated each incoming volume, controlling for mean signal and linear trends. For each volume, we subtracted the expected signal intensity based on the GLM from the measured signal intensity to discount nuisance effects (e.g., low-frequency signal drifts). The residual reflected BOLD signal fluctuations and other unmodeled fMRI noise. The residual was further normalized by the average GLM residual over the first 25 functional images of the baseline to yield an estimated activation strength at each voxel at time *t* in units of standard deviation.

### 2.6. Session 3

Session 3 occurred 33.9 ± 59.3 days after Session 2. Two participants (one in each condition) had a long lapse between sessions (>200 days) due to COVID-19. Excluding these two participants, Session 3 occurred 16.2 ± 10.2 days after Session 2.

#### 2.6.1. AH Assessment

Participants were administered the PSYRATS at the beginning of the session and underwent a scan that was unrelated to the current analyses.

### 2.7. Data Analysis

#### 2.7.1. Preprocessing

(1) T1 sMRI was processed via FreeSurfer version 7.0 by calculating the distance between the gray–white matter boundary and the pial surface across the entire cerebral cortex [67]. In accordance with a standardized quality assurance protocol, the reconstructed cortical surface was manually examined for accuracy and edited when necessary. (2) dMRI was preprocessed using FreeSurfer's TRACULA [68], which included calculating measures of dMRI scan head motion, intrasubject registration (individual dMRI to individual T1), intersubject registration (individual to a common template space), tensor fitting for extraction of tensor-based FA measure, and using TRACULA's manually annotated training subjects to calculate anatomical priors for white-matter pathways. Bayesian estimation of diffusion parameters obtained using sampling techniques for modeling crossing fibers (bedpostx) was then used on preprocessed dMRI data to determine the number of crossing fibers per voxel and model these fibers [68]. (3) rsfMRI preprocessing was performed using *fMRIPrep* 21.0.0 [69]. We applied realignment, coregistration, normalization, susceptibility distortion correction, segmentation of gray matter, white matter, cerebrospinal fluid (CSF) tissues, skull stripping, and confounds extraction. We imported the preprocessed data and confounds into CONN Toolbox v20.b [70] and further performed smoothing with a 6 mm kernel. Motion outliers were defined as volumes with global signal *z* > 3 and framewise displacement >0.5 mm using artifact detection tools (ARTs, www.nitrc.org/projects/artifact_detect) [71]. We used the aCompCorr method [72] to identify the principal components of noise from white matter and CSF. We applied nuisance regression (five white matter components, five CSF components, 12 realignment parameters [three translation, three rotation, and their first derivatives], linear drift and its first derivative effect, and motion outliers), and band-pass filtering (0.008–0.09 Hz).

#### 2.7.2. Cortical Thickness Analysis

We used Freesurfer's GLM (mri_glmfit) to estimate vertices whose thickness correlated with AH change and then extracted clusters (mri_surfcluster; *p* < 0.05) within a combined binary mask of STG (bilateral anterior and posterior STG regions of the Harvard-Oxford 25% probability atlas) and DLPFC (DLPFC node of the CEN derived from an independent component analysis of the Human Connectome Project dataset, *N* = 497; [73] ROIs. One cluster in the right STG emerged as the largest cluster (372.29 mm^2^; other smaller clusters had less than 50 mm^2^). We converted this STG cluster into each subject's space (mri_label2label) and extracted the mean cortical thickness of all vertices within this cluster (mri_anatomical_stats).

#### 2.7.3. FA Analysis

We used TRACULA's pointwise assessment of streamlined tractography attributes [74] to calculate tract statistics for 16 tracts: bilateral AF, dorsal cingulum bundle (dCB), SFL, and CC (body, genu, rostrum, and splenium). We fit a GLM at each point along each tract (mri_glmfit) with FA as the independent variable and AH change as the dependent variable. We then performed cluster-wise correction for multiple comparisons (mri_glmfit-sim) using a permutation simulation that ran 10,000 times [75]. The cluster-defining threshold was *p*  < 0.05.

#### 2.7.4. Functional Connectivity Analysis

Using the CONN toolbox [70], we performed functional connectivity analyses using a subject-specific DMN ROI (see 2.4.3.*: DMN and MC Functional Localizer*). We searched the whole brain for voxels whose connectivity with the seed region at baseline correlated with PSYRATS score change, controlling for framewise displacement [63], and were evaluated at *p* < 0.05 (uncorrected).

#### 2.7.5. Model Comparisons

We conducted model comparison analyses using only baseline imaging metrics that were individually associated with AH change as independent variables. Specifically, we conducted a series of hierarchical multiple regression analyses using IBM SPSS Statistics (Version 28) to test whether baseline AH score, sMRI feature (right STG thickness), dMRI feature (dCB FA), and rsMRI features (DMN–PCC and DMN–DLPFC functional connectivity) each explained additional unique variance in AH change, producing four models with AH change as the dependent variable.

## 3. Results

### 3.1. AH Change Following NFB

As reported in previous papers based on the same study [25, 61], participants who received real NFB reported significantly decreased AH after NFB (*t* [11] = 2.30, *p*=0.042, two-tailed, paired-sample *t*-test). There was no significant interaction when we ran an ANOVA test on AH change contrasting real vs. sham groups.

### 3.2. Baseline AH Correlated With AH Change

**Figure 2:**
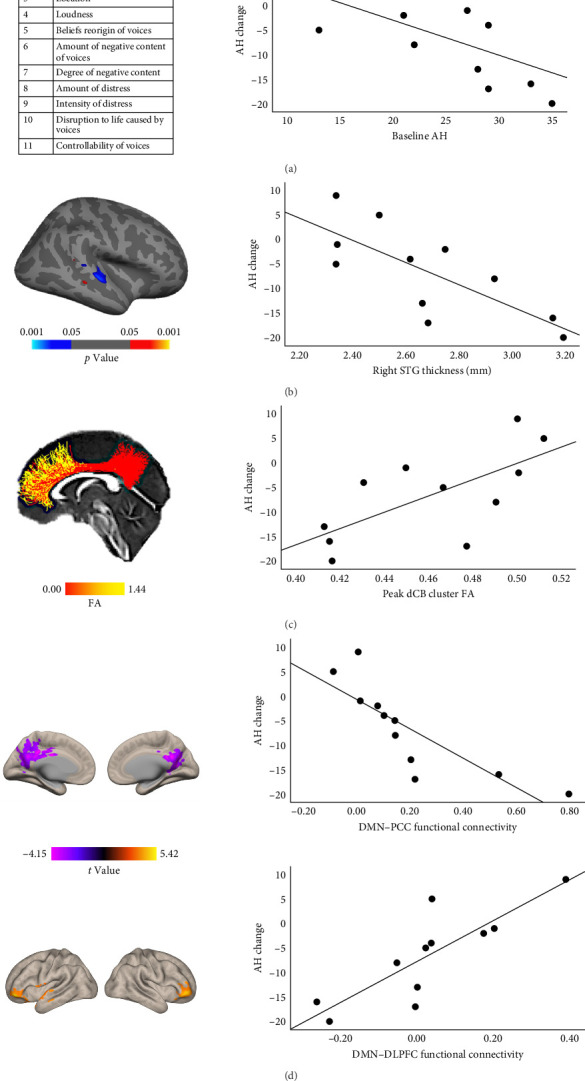
Multimodal neuroimaging features were associated with AH score change. (A) Greater AH reduction was associated with higher baseline AH. Eleven items on the PSYRATS auditory hallucination subscale were used to score AH. (B) Greater AH reduction was associated with greater right STG thickness (*p* < 0.05, uncorrected). The left panel shows the correlation between cortical thickness at each vertex and AH change. (C) Greater AH reduction was associated with lower dCB integrity (*p* < 0.05, corrected). The left panel shows the correlation between FA at each vertex and AH change (*q*_FDR_ < 0.05). (D) Greater AH reduction was associated with stronger DMN–PCC resting-state functional connectivity and stronger DMN–DLPFC anticorrelation (i.e., negative connectivity) (*p* < 0.05, uncorrected). The left panel shows the correlation between resting-state functional connectivity with individualized DMN seed at each voxel and AH change. AH, auditory hallucination; dCB, dorsal cingulum bundle; DLPFC, dorsolateral prefrontal cortex; DMN, default mode network; FA, fractional anisotropy; PCC, posterior cingulate cortex; PSYRATS, psychotic symptom rating scales; STG, superior temporal gyrus.

In the real NFB group, baseline PSYRATS score was marginally correlated with AH change (*r* = −0.59, *p*=0.058), meaning greater baseline AH severity was associated with greater AH reduction (Figure 2A).

### 3.3. Baseline Cortical Thickness in the Right STG Correlated With AH Score Change

In the real NFB group, vertexwise analysis of cortical thickness within the STG and DLPFC identified one large cluster in the right STG with a significant negative correlation with AH change (*p* < 0.05; Figure 2B).

### 3.4. Baseline FA in Dorsal CB Correlated With AH Score Change

In the NFB real group, average FA within the dCB was significantly positively correlated with AH change (*r =* 0.68, *p*=0.022) and survived *q*_FDR_ <0.05. Further pointwise analysis of FA within the dCB found one significant cluster with a positive correlation with AH change (*p*  < 0.05, corrected using permutation simulations; Figure 2C).

### 3.5. Baseline DMN Functional Connectivity Correlated With AH Score Change

In the real NFB group, we found that subject-specific DMN connectivity to bilateral PCC and DLPFC was significantly negatively correlated with AH change (*p* < 0.05, uncorrected; Figure 2D). More improvement in AH post-NFB was thus associated with (1) stronger positive connectivity within the DMN (DMN–PCC) as well as (2) stronger anticorrelation (i.e., more negative connectivity) between DMN and bilateral DLPFC.

### 3.6. Baseline AH, Cortical Thickness, and Functional Connectivity Uniquely Predicted AH Change

To test whether neuroimaging markers of cortical thickness, white matter integrity, and functional connectivity explained significantly more variance in AH change than baseline AH alone, we ran a series of hierarchical linear regression analyses with AH score change as the dependent measure, using baseline AH score, sMRI features, dMRI feature, and rsMRI features as independent variables. Baseline AH score alone accounted for 34.4% of the variance in AH change (*F* [1,9] = 4.71, *p*=0.058; total adjusted *R*^2^ = 0.271). Compared to baseline AH alone, right STG thickness significantly explained an additional 26.1% of the variance in AH change (*F* [1,8] = 5.29, *p*=0.050; total adjusted *R*^2^ = 0.506), DMN–PCC connectivity significantly explained an additional 34.3% of the variance in AH change (*F* [1,8] = 8.73, *p* < 0.018; total adjusted *R*^2^ = 0.608), and DMN–DLPFC connectivity significantly explained an additional 46.4% of the variance in AH change (*F* [1,8] = 19.24, *p*=0.002; total adjusted *R*^2^ = 0.759). We dropped the dMRI feature as it did not significantly add explained variance on top of the regression model with baseline AH. We combined baseline AH, right STG thickness, and DMN–DLPFC connectivity into the same regression model as independent variables to test contributions by each modality and found that only DMN–DLPFC connectivity significantly explained unique variance in AH change (*β* = 0.80, *p*=0.045). Remarkably, DMN–DLPFC connectivity alone significantly explained 69.1% of the variance in AH change (*F* [1,9] = 20.10, *p*=0.002; total adjusted *R*^2^ = 0.65.6). A full correlation matrix, including pairwise correlations among all predictor variables, can be found in Table 1.

## 4. Discussion

AH in SZ involves complex neural circuitries that underlie auditory and cognitive processing. Our network-based NFB approach leverages the connectedness between these circuitries to modulate them and improve symptoms. In this study, we took a multimodal neuroimaging approach, which included MRI, dMRI, and rsfMRI, to examine potential baseline predictors of NFB success for individuals with SZ. Results revealed that a reduction in AH score was individually associated with a higher baseline AH score, greater STG thickness, decreased dCB FA, increased within-DMN resting-state functional connectivity, and increased DMN–DLPFC anticorrelation. In a combined model, DMN–DLPFC connectivity significantly explained unique variance in AH change. These results suggest that a specific rsMRI measure, namely DMN–DLPFC connectivity, may be a promising predictor of NFB success.

We hypothesized that a thinner cortex in the STG would predict larger reductions in AH as it has been shown to be associated with greater AH severity [28–30] and better treatment response [31]. Contrary to our hypothesis, larger reductions in AH were found to be associated with thicker STG at baseline and explained unique variance in AH change over and beyond baseline AH. The specific correlation between AH change and STG cortical thickness suggests that more computational capacity within the STG may enable greater learning during NFB to appropriately downregulate STG activity and better process signals from control regions to reduce source misattribution [76, 77].

We also hypothesized that reduced integrity in tracts connecting auditory processing and comprehension areas would predict larger reductions in AH and specifically found that reduced left dCB integrity was associated with greater AH reduction. This is consistent with previous literature showing that lower cingulum FA values are associated with AH in SZ [35, 42] and with antipsychotic treatment response and positive symptom reduction in SZ [47]. Given the significant negative correlation between baseline dCB FA and baseline AH, our results may indicate that individuals with less integrity in the cingulum at baseline have more room to improve post-intervention. Importantly, consistent with observations that CB changes are part of a wide network of disrupted limbic/memory pathways and are not unique to SZ [78, 79], we also found lower dCB FA was associated with stronger within-DMN connectivity.

We hypothesized and found that increased DMN–PCC connectivity and increased DMN–DLPFC anticorrelation were associated with larger reductions in AH. The associations with AH reduction are consistent with previous evidence of alterations in within-DMN connectivity and DMN CEN interactions in AH [80]. DMN hyperconnectivity in SZ has been related to the misattribution of internal thoughts to an external source in those with AH [49, 50]. In our study, individuals with higher baseline within-DMN connectivity had more severe AH at baseline and, therefore, may have more room for improvement post-NFB. In our sample of subjects receiving real NFB, we also observed reduced DMN connectivity both during the NFB task and during rsfMRI post-NFB [58], suggesting that rewiring of the functional architecture within the DMN may be important for effective NFB even when STG was the primary modulation target. DMN–DLPFC anticorrelation is reduced in SZ, reflecting reduced task-related suppression [15] and cognitive impairment [52]. Nevertheless, our findings indicate that among individuals with SZ, those with stronger anticorrelations at baseline showed more improvement in AH post-NFB, possibly due to their relatively preserved cognitive capacity to appropriately direct attentional resources and integrate feedback. The implication of the DLPFC is consistent with our observations of sustained DLPFC activation and reduced STG-DLPFC connectivity during NFB [56], indicating that the DLPFC plays an instrumental role in modulating attention to auditory events. These results also suggest that more emphasis should be placed on modulating distributed networks such as the DMN (especially its medial components) and CEN for the treatment of AH. In contrast, repetitive transcranial magnetic stimulation, another noninvasive neuromodulation technique, has predominantly targeted the temporo-parietal junction (TPJ) and shown limited success [81, 82]. Importantly, beyond psychosis, within-DMN connectivity and DMN–DLPFC connectivity are implicated in domain-general, transdiagnostic processes [83, 84] such as self-referential processing [85, 86], rumination [87], and executive control [88, 89]. Therefore, DMN functional connectivity may serve as a treatment target and predictor in other disorders such as depression and anxiety [90, 91].

Last but not least, when we combined all the independent variables (baseline AH, sMRI, and rsfMRI measures) in the same regression model, DMN–DLPFC connectivity explained unique variance in AH change. The prominent contribution of DMN connectivity agrees with existing literature that points to DMN connectivity as a predictor of treatment response [53–55]. Our findings also suggest that, while within-DMN connectivity explained variations in symptom change via its association with baseline symptom severity, DMN–DLPFC connectivity presented a separate dimension that may contribute to NFB success via cognitive control mechanisms, especially as NFB can be considered a form of reinforcement learning involving processes such as representation and manipulation of mental states [92]. Importantly, in this study, personalized DMN masks derived from a baseline resting-state scan were used as an ROI in the resting-state analysis. The individualized functional localization method may have contributed to the unique advantage of a rsfMRI-based marker. In our study, it is possible that functional correlates (e.g., derived from fMRI) outperformed structural correlates (e.g., derived from sMRI or dMRI) as they better capture information flow and index cognitive flexibility [93], especially in the DMN and CEN [94, 95].

Due to practical constraints such as the COVID-19 pandemic and stringent inclusion criteria (e.g., severe AH), the current study had a small sample size and, therefore, was limited in generating accurate effect sizes or detecting less robust effects. In addition, we assessed AH only at a one-time point post-NFB and, therefore, could not establish a symptom change trajectory. Future research should also systematically examine activation patterns during relevant baseline tasks, such as the STG localizer task [25, 56] or self-reference task [57], as potential predictors for NFB success as they may reflect specific dimensions of AH symptomatology. Nevertheless, as NFB may offer a noninvasive approach to treat AH, future research should attempt to replicate current findings as well as more deeply sample within individuals to further increase precision in who and when individuals should receive NFB treatment.

## Figures and Tables

**Table 1:** Correlations between all tested variables.

Baseline feature	AH change	Baseline AH	STG thickness	dCB FA	DMN–PCC RSFC
Baseline AH	−0.59	—	—	—	—
STG thickness	−0.75*⁣*^*∗∗*^	0.57	—	—	—
dCB FA	0.68*⁣*^*∗*^	−0.71*⁣*^*∗∗*^	−0.48	—	—
DMN–PCC RSFC	−0.83*⁣*^*∗∗*^	0.67*⁣*^*∗*^	0.83*⁣*^*∗∗*^	−0.68*⁣*^*∗*^	—
DMN–DLPFC RSFC	0.83*⁣*^*∗∗*^	−0.32	−0.82*⁣*^*∗∗*^	0.59	−0.79*⁣*^*∗∗*^

*Note:* All values are Pearson's correlation coefficients. *p*=two-tailed significance.

Abbreviations: AH, auditory hallucinations; dCB, dorsal cingulum bunble; DLPFC, dorsolateral prefrontal cortex; DMN, default mode network; FA, fractional anisotropy; PCC, posterior cingulate cortex; RSFC, resting state functional connectivity; STG, superior temporal gyrus.

*⁣* ^*∗*^ *p* < 0.05.

*⁣* ^*∗∗*^ *p* < 0.01.

## Data Availability

The data that support the findings of this study are available from the corresponding author upon reasonable request.
